# Different effects of heating and freezing treatments on the antioxidant properties of broccoli, cauliflower, garlic and onion. An experimental in vitro study

**DOI:** 10.1590/1516-3180.2019.004406082019

**Published:** 2019-11-07

**Authors:** Hikmet Can Çubukçu, Nazlı Seda Durak Kılıçaslan, İlker Durak

**Affiliations:** I MD. Medical Biochemistry Specialist, Department of Medical Biochemistry, Mareşal Çakmak Devlet Hastanesi, Erzurum, Turkey.; II MSc. Research Assistant, Department of Field Crops, Ankara Üniversitesi Ziraat Fakültesi, Ankara, Turkey.; III PhD. Professor, Department of Medical Biochemistry, Ankara Üniversitesi Tıp Fakültesi, Ankara, Turkey.

**Keywords:** Brassica, Garlic, Onions, Antioxidants

## Abstract

**BACKGROUND:**

Vegetables have some beneficial effects on human health due to their antioxidant compounds, like polyphenols. Cooking leads to many physical and chemical changes to plant structure that can alter the phytochemical compounds of vegetables.

**OBJECTIVES:**

To investigate the effects of heat treatment and freezing on the antioxidant properties of garlic, onion, broccoli and cauliflower.

**DESIGN AND SETTING:**

Experimental in vitro study in a university laboratory.

**METHODS:**

Fresh broccoli (*Brassica oleracea* var. italica), cauliflower (*Brassica oleracea* var. botrytis), garlic (*Allium sativum*) and onion (*Allium cepa*) were obtained from a local store. These vegetables were divided into three treatment groups: raw, heated and frozen. The heat treatment consisted of heating them in a drying oven at 150 °C for 20 minutes. The freezing treatment consisted of keeping them frozen at -20 °C until analysis. The total phenolic content, antioxidant activity and malondialdehyde levels of the vegetables were measured using the Folin-Ciocalteu phenol reagent, 2,2-diphenyl-1-picrylhydrazyl radical scavenging activity and thiobarbituric acid reactive substances, respectively.

**RESULTS:**

Heat treatment had deleterious effects on the antioxidant properties of onion and garlic; and it decreased the antioxidant activity of broccoli. Freezing improved the antioxidant activity of broccoli and garlic, but had detrimental effects for cauliflower and onion.

**CONCLUSIONS:**

Heat treatment and freezing exhibit different effects on the antioxidant properties of broccoli, cauliflower, garlic and onion. Convenient cooking and storage patterns should be identified for each vegetable, to obtain the best nutritional benefit from the antioxidant compounds of vegetables.

## INTRODUCTION

Consumption of fruits and vegetables is associated with reduced risks of hypertension, stroke, coronary heart disease, cancer, dementia and type 2 diabetes mellitus.^1^ The beneficial effects of vegetables and fruits are attributed to presence of certain bioactive compounds, like polyphenols. Flavonoids, phenolic acids, lignins and stilbenes are polyphenols that exhibit antioxidant effects.^2^

Broccoli (*Brassica oleracea* var. italica) is a member of the Cruciferous family.^3^ Glucosinolates, flavonoids, cinnamic acid derivatives, carotenoids, ascorbic acid, xanthophylls and minerals are among the substances that broccoli contains. The anticarcinogenic, antimutagenic and antioxidant properties of these compounds contribute to the health benefits from broccoli.^4^

Cauliflower (*Brassica oleracea* var. botrytis L.) is also included in the Cruciferous family. The components of cauliflower include glucosinolates, ascorbic acid, carotenoids, phenolic compounds and vitamin E.^3^ It has anticarcinogenic and antioxidant effects, like other cruciferous vegetables.^5^^,^^6^

Garlic (*Allium sativum*) has been consumed as a vegetable and natural remedy for centuries.^7^ In addition to organosulfur compounds, garlic contains high amounts of vitamins, minerals and phenolic compounds. So far, garlic has been reported to have anticancer, antimicrobial, antioxidant, anti-inflammatory, immunomodulatory and cardioprotective properties.^8^

Onion (*Allium cepa* L.) is cultivated in many parts of the world, given its adaptable nature. Flavonoids and alk(en)yl cysteine sulfoxides are the main bioactive groups in onion, and these compounds are responsible for antithrombotic, antiasthmatic, anticarcinogenic, antioxidant, antifungal and antibacterial effects.^9^

Most vegetables are consumed after a cooking procedure that may involve boiling, microwaving, steaming or baking. Cooking can lead to many physical and chemical changes in plant structure. The concentration of phytochemical compounds in vegetables can be increased through the matrix softening effect and improved extractability. Conversely, heat treatment can cause thermal degradation of these nutrients.^10^ The antioxidant activity of vegetables is mainly based on their phytochemical compounds, like polyphenols. Cooking can trigger not only oxidation of these compounds, but also leakage of water-soluble compounds. Nevertheless, inactivation of prooxidant enzymes through heat treatment can result in enhanced antioxidant activity.^11^ Additionally, partially oxidized polyphenols can have higher antioxidant activity than the non-oxidized form. Moreover, heat treatment can generate more potent antioxidant products called Maillard reaction products.^12^

Fresh vegetables are preferably stored in a refrigerator or freezer because they only remain fresh for a short time. However, freezing can also impair the nutritional quality of some vegetables.^13^

## OBJECTIVE

The aim of this study was to investigate the effects of heat treatment and freezing on the antioxidant properties of garlic, onion, broccoli and cauliflower by analyzing total phenolic content, antioxidant activity and malondialdehyde levels.

## METHODS

### Chemicals used

Methanol, ethanol, trichloroacetic acid, disodium hydrogen phosphate, potassium dihydrogen phosphate and sodium carbonate were purchased from Merck (Darmstadt, Germany). 2-thiobarbituric acid, 2,2-diphenyl-1-picrylhydrazyl, Folin-Ciocalteu phenol reagent and gallic acid were purchased from Sigma-Aldrich (Steinheim, Germany).

### Vegetables and sample preparation

Fresh broccoli (*Brassica oleracea* var. italica), cauliflower (*Brassica oleracea* var. botrytis), garlic (*Allium sativum*) and onion (*Allium cepa*) were obtained from a local store. Each vegetable was divided into three treatment groups: raw, heated and frozen.

The heat treatment consisted of heating the vegetables in a drying oven at 150 °C for 20 minutes. After the heat treatment, the vegetables were stored at 4 °C for 12 hours in contact with air until analysis. The freezing treatment consisted of keeping the vegetables frozen at -20 °C until the time of analysis. The raw vegetables consisted of fresh vegetables stored at 4 °C in a sterile container in the refrigerator. A sample of 10 grams of each vegetable was used for analysis.

After heating and freezing treatment, the vegetables were homogenized in distilled water using the DIAX 900 homogenizer (Heidolph, Kelheim, Germany) just before analysis, to make an aqueous extract at a concentration of 10% (w/v). After centrifugation at 4000 g for 10 minutes, the supernatant fractions of the homogenates were isolated for analysis.

### Measurement of malondialdehyde levels

Malondialdehyde (MDA) levels were measured spectrophotometrically by using the thiobarbituric acid reactive substances (TBARS) method. This is based on the interaction between thiobarbituric acid (TBA) and MDA in an acidic solution to yield a pink-colored dye.^14^ One ml of a solution of ethanol (95%; v/v), phosphate buffer, trichloroacetic acid (20%; w/v) and thiobarbituric acid (2%; w/v) was added to each 0.1 ml of the sample in a tube. After 30 minutes of incubation in boiling water, the tubes were centrifuged at 4 °C in a Harrier 18/80 centrifuge (MSE, London, UK). The absorbances of the clear supernatant fractions were measured spectrophotometrically at 532 nm by means of a Helios Alpha ultraviolet/visible spectrophotometer (Unicam, Cambridge, UK).

### Analysis of antioxidant activity

The antioxidant activity levels of the vegetables were determined according to their 2,2-diphenyl-1-picrylhydrazyl (DPPH) radical scavenging activity.^15^ 975 µl of methanolic DPPH solution was added to each 25 µl sample in a tube. Following incubation for 30 minutes in the dark, the decline in absorbance was measured at 517 nm spectrophotometrically, against a negative control. Antioxidant activity was expressed as the percentage DPPH depletion.^16^

### Determination of total phenolic content

The total phenolic content of the vegetables was determined using the Folin-Ciocalteu phenol reagent, as described by Obanda and Owuor.^17^ Briefly, 0.5 ml of Folin-Ciocalteu phenol reagent was mixed with 0.5 ml of the aliquot. After incubation for 5 minutes, 1 ml of Na_2_CO_3_ and 1 ml of water were added to the mixture. Following a further 30 minutes of incubation, the blue-colored end product was measured spectrophotometrically at 700 nm.

### Statistical analysis

The data analyzed in this study were obtained from ten repetitions and were evaluated using the Statistical Package for the Social Sciences (SPSS), version 11.5. Multiple comparisons of the experimental groups were analyzed using the ANOVA and Kruskal-Wallis tests. Tukey’s honestly significant difference (HSD), Tamhane’s and Dunn’s tests were used for post-hoc evaluation of subgroups, where suitable. The relationships between biochemical parameters were assessed using Spearman and Pearson correlation tests. The statistical significance level was taken to be P values less than 0.05.

## RESULTS

The freezing and heat treatments had different effects on the antioxidant activity, phenolic content and MDA levels of the vegetables, as shown in Table 1 and Figures 1-4. All treatments were compared with raw vegetables.

**Table 1.** Comparisons of the 2,2-diphenyl-1-picrylhydrazyl (DPPH) radical scavenging activity, phenolic compounds and malondialdehyde levels of raw, frozen and heated vegetables

Vegetable	Test	Group	n	Mean	SD	ANOVA - KW P-values	Post-hoc comparison P-values			Tests used
							raw	frozen	heated	
Broccoli	DPPH radical scavenging activity (%)	raw	10	15.47	1.25	< 0.001		< 0.001	0.002	ANOVA-Tukey’s HSD
		frozen	10	19.93	1.43		< 0.001		< 0.001	
		heated	10	17.52	0.72		0.002	< 0.001		
	Phenolic compounds (mg/dl)	raw	10	6.41	0.13	< 0.001		0.017	0.112	KW-Dunn’s Test
		frozen	10	8.12	0.10		0.017		< 0.001	
		heated	10	6.21	0.13		0.112	< 0.001		
	Malondialdehyde (nmol/g)	raw	10	47.74	11.54	0.018		0.015	0.152	ANOVA-Tukey’s HSD
		frozen	10	60.65	10.12		0.015		0.526	
		heated	10	55.97	6.24		0.152	0.526		
Cauliflower	DPPH radical scavenging activity (%)	raw	10	4.38	0.71	0.048		0.042	0.687	ANOVA-Tukey’s HSD
		frozen	10	3.73	0.49		0.042		0.214	
		heated	10	4.17	0.47		0.687	0.214		
	Phenolic compounds (mg/dl)	raw	10	3.87	0.08	< 0.001		0.072	< 0.001	ANOVA-Tukey’s HSD
		frozen	10	4.00	0.15		0.072		< 0.001	
		heated	10	2.91	0.12		< 0.001	<0.001		
	Malondialdehyde (nmol/g)	raw	10	56.46	7.40	0.022		0.021	0.110	ANOVA-Tukey’s HSD
		frozen	10	69.20	9.35		0.021		0.717	
		heated	10	65.73	12.35		0.110	0.717		
Garlic	DPPH radical scavenging activity (%)	raw	10	30.59	2.15	< 0.001		< 0.001	< 0.001	ANOVA-Tukey’s HSD
		frozen	10	39.72	2.15		< 0.001		< 0.001	
		heated	10	17.69	2.24		<0.001	< 0.001		
	Phenolic compounds (mg/dl)	raw	10	12.96	0.20	< 0.001		0.996	< 0.001	ANOVA-Tukey’s HSD
		frozen	10	12.95	0.20		0.996		< 0.001	
		heated	10	9.11	0.21		< 0.001	< 0.001		
	Malondialdehyde (nmol/g)	raw	10	739.40	95.87	0.595		na	na	ANOVA-Tukey’s HSD
		frozen	10	753.27	142.26		na		na	
		heated	10	703.51	89.11		na	na		
Onion	DPPH radical scavenging activity (%)	raw	10	13.72	0.75	< 0.001		< 0.001	< 0.001	ANOVA-Tamhane’s Test
		frozen	10	9.55	0.74		< 0.001		< 0.001	
		heated	10	6.04	0.19		< 0.001	< 0.001		
	Phenolic compounds (mg/dl)	raw	10	7.18	0.13	< 0.001		< 0.001	< 0.001	ANOVA-Tamhane’s Test
		frozen	10	4.50	0.05		< 0.001		< 0.001	
		heated	10	4.72	0.04		< 0.001	< 0.001		
	Malondialdehyde (nmol/g)	raw	10	51.78	13.55	< 0.001		0.362	< 0.001	ANOVA-Tukey’s HSD
		frozen	10	60.97	15.17		0.362		< 0.001	
		heated	10	91.05	15.67		< 0.001	< 0.001		

SD = standard deviation; ANOVA = analysis of variance; KW = Kruskal-Wallis test; HSD = honestly significant difference; na = not available.

Although the freezing treatment increased the antioxidant activity levels of broccoli and garlic, those of cauliflower and onion declined after this treatment. The phenolic contents of broccoli and cauliflower were enhanced through freezing, while the phenolic content of onion was reduced. Nevertheless, the MDA levels of frozen vegetables were found to be higher than those of raw vegetables.

Even though the heat treatment increased the antioxidant activity of broccoli, the antioxidant activity levels of heated garlic and onion were found to be lower than those of raw garlic and onion. Furthermore, the phenolic content of garlic, onion and cauliflower decreased through the heat treatment. Although the MDA concentration in onion was increased through the heat treatment, there were no statistically significant differences between raw and heated broccoli, cauliflower and garlic.

In addition to the low positive correlation between the phenolic content and antioxidant activity levels of onion (r = 0.467, P < 0.01) and broccoli (r = 0.412, P < 0.05), a moderate positive correlation was observed between the phenolic content and antioxidant activity of garlic (r = 0.639, P < 0.01). Even though the antioxidant activity levels of onion (r = -0.644, P < 0.01) and cauliflower (r = -0.381, P < 0.05) showed negative correlations with their MDA levels, the antioxidant activity of broccoli showed a significantly low positive correlation with MDA level (r = 0.422, P < 0.05), as shown in Table 2.

**Table 2.** Correlations between the 2,2-diphenyl-1-picrylhydrazyl (DPPH) radical scavenging activity, phenolic compounds and malondialdehyde levels of vegetables

Vegetable	Test	Correlation coefficient (r)		
		DPPH radical scavenging activity (%)	Phenolic compounds (mg/dl)	Malondialdehyde (nmol/g)
Broccoli	DPPH radical scavenging activity (%)		0.412*	0.422*
	Phenolic compounds (mg/dl)	0.412*		0.309
	Malondialdehyde (nmol/g)	0.422*	0.309	
Cauliflower	DPPH radical scavenging activity (%)		-0.200	-0.381*
	Phenolic compounds (mg/dl)	-0.200		-0.022
	Malondialdehyde (nmol/g)	-0.381*	-0.022	
Garlic	DPPH radical scavenging activity (%)		0.639**	0.115
	Phenolic compounds (mg/dl)	0.639**		0.112
	Malondialdehyde (nmol/g)	0.115	0.112	
Onion	DPPH radical scavenging activity (%)		0.467**	-0.644**
	Phenolic compounds (mg/dl)	0.467**		-0.254
	Malondialdehyde (nmol/g)	-0.644**	-0.254	

*P < 0.05; **P < 0.01.

## DISCUSSION

The effects of heat treatment on broccoli, cauliflower, onion and garlic were investigated in the present study. The heat treatment that was applied was analogous to baking.

The present study revealed that heat treatment led to an increase in the antioxidant activity of broccoli, while its total phenolic content was stable during this process (P = 0.002 and P = 0.112 respectively). Additionally, the malondialdehyde levels remained unchanged during the treatment.

The cooking methods on this topic that were previously studied have mainly approximated boiling, microwaving and steaming. Pellegrini et al. reported that boiling and oven-steaming caused increases in the total phenolic content and antioxidant capacity of fresh broccoli, while microwaving did not.^18^ In contrast to these findings, Zhang and Hamauzu indicated that microwaving and boiling both led to reduction of the total phenolic content, total carotenoids, ascorbic acid levels and antioxidant activity of fresh broccoli.^19^ To our knowledge, the impact of baking on broccoli had not previously been investigated. The heat treatment used in the present study is commonly used in baking. The increased antioxidant activity established in the present study may have been due to enhanced extractability of antioxidants other than phenolics, like ß-carotene. This is in line with data from a previous study that demonstrated that the ß-carotene isomer was released through cooking.^20^

The contradictory data in the literature regarding cauliflower mainly focused on certain cooking methods, including boiling, steaming, microwaving and sous vide (cooking under vacuum). Volden et al. noticed that boiling, blanching and steaming had all detrimental effects on total phenolic content, ascorbic acid levels and antioxidant activity.^21^ Reis et al. stated that while microwaving had no effect on the phenolic content of cauliflower, boiling, steaming and sous vide led to reductions of phenolic compounds. Additionally, antioxidant activity was only increased through microwaving.^22^ Girgin and El demonstrated that steaming caused increases in total phenolic content and antioxidant activity, but that boiling caused reduced levels.^23^

As far as we know, the present study is the first to examine the effects of baking on cauliflowers’ antioxidant properties. Heated cauliflower showed lower total phenolic content than raw cauliflower, as shown in Table 1. However, the antioxidant activity of cauliflower seems to be more stable, possibly because of great retention of antioxidant compounds other than phenolic compounds.

We demonstrated that heat treatment had a reducing effect on the phenolic content and antioxidant activity of garlic. Likewise, Park et al. noticed that the DPPH radical scavenging activity and total phenolic content of raw garlic extract were higher than those of heated garlic extract.^24^ A positive relationship between the phenolic content and antioxidant activity of garlic was found in our study (r = 0.639, P < 0.01). Hence, it could be hypothesized that deterioration of antioxidants, including phenolic compounds, led to the decrease in antioxidant activity.

In our study, the total phenolic content of onion was decreased through heat treatment. To date, contradictory results regarding alteration of phenolic content have been reported in the literature. Although microwave heating was found to increase the concentration of quercetin, possibly through facilitating extractability, reduced flavonoid content was observed in boiled onion, possibly due to transition to water.^25^ Quercetin glycosides, which are the main flavonoids of onion, can undergo thermal degradation. Rohn et al. showed that quercetin diglucosides were converted to quercetin monoglucosides and quercetin aglycone through roasting.^26^

Reduced flavonoid content in onion was detected when frying, sautéing, boiling, steaming and microwaving treatments were applied. However, baking for 5 minutes at 200 °C did not show any effects on the flavonoid content of onion, as indicated in the same study.^27^ On the contrary, the quercetin diglucoside and quercetin glucoside concentrations in onion were decreased through heat treatment that was maintained for longer than 10 minutes.^28^ The discrepancy might be attributable to the variety of heat treatment, the degree of applied heat and its duration. Our results are in agreement with the study by Harris et al.^28^ Not only the phenolic content but also the antioxidant activity of onion decreased through heat treatment (Table 1). Furthermore, a positive correlation was observed between the phenolic content and antioxidant activity (Table 2). These results might be explained by the fact that the antioxidant activity of onion is attributable to its phenolic content. Additionally, the increase in malondialdehyde levels showed that the oxidation process induced by heat treatment led to degradation of antioxidant ingredients.

The effects of consumer storage conditions on broccoli, cauliflower, garlic and onion were investigated in the current study. It should be borne in mind that the group of raw vegetables was stored in a refrigerator at 4 °C to mimic consumer routines.

Frozen broccoli was found to contain higher antioxidant activity, total phenolic content and malondialdehyde levels, compared with raw broccoli. According to our findings, frozen storage preserves the antioxidant compounds of broccoli better than does raw storage.

Divergent results have been reported in the literature. Li et al. stated that the trans beta-carotene and folate levels of broccoli decreased through freezing, while the ascorbic acid levels did not change.^29^ Patras et al. indicated that the antioxidant activity and ascorbic acid levels of unblanched frozen broccoli were lower than those of fresh and blanched frozen broccoli. No significant difference in phenol concentration was observed between the study groups.^30^ Conversely, slightly increased phenolic content was reported for two broccoli cultivars. The total carotenoid concentrations of frozen broccoli cultivars were higher than those of raw ones.^31^ These conflicting findings may have been due to variation in the cultivars studied. Furthermore, vegetables were blanched prior to freezing in some studies.

Frozen cauliflower showed lower antioxidant activity in the present study. Moreover, its malondialdehyde levels increased with freezing. However, no significant change was detected in its total phenolic content. These findings might be explained by the reduction in antioxidants through freezing. These decreased antioxidant levels would thus be due to decrease in the levels of some compounds other than phenolics. Murcia et al. showed that freezing did not significantly affect the antioxidant capacity of cauliflower.^32^ Additionally, Li et al. did not find any statistically significant differences in trans beta-carotene and ascorbic acid levels between frozen and raw cauliflower.^29^ In contrast with previous studies, Volden et al. reported that total phenolic content, L-ascorbic acid levels and antioxidant capacities of some cauliflower cultivars were reduced through freezing.^33^ Our results are partially in agreement with the latter study. In addition to decreased antioxidant activity and increased malondialdehyde levels, we also observed a negative correlation between these two parameters (r = -0.381). It can be inferred from our findings that freezing may somehow lead to an oxidation process in cauliflower.

Frozen storage maintained the antioxidant activity of garlic better than raw storage. However, the freezing process did not give rise to any change in total phenolic content or malondialdehyde levels. Thus, the increased antioxidant activity could be attributable to preservation of antioxidants other than phenolic compounds. Murcia et al. claimed that freezing had a detrimental effect on the antioxidant activity of garlic. However, in Murcia’s study, frozen vegetables were separately purchased from a supermarket as commercially processed vegetables.^32^ Therefore, the low quality of the study design makes it hard to come to an accurate conclusion.

In the present study, the phenolic content and antioxidant activity of frozen onion were found to be lower than those of raw onion. On the other hand, the malondialdehyde concentration did not change through freezing, as shown in Table 1. Nimfali and Bacchiocca found that the oxygen radical absorbance capacity and phenolic content were higher in frozen onion than in raw onion. Nevertheless, according to those authors, addition of ascorbic acid for preservation of the packaged product interfered with the analysis.^34^ In contrast to the previous study, our results showed that frozen storage did not possess the essential conditions for preserving the antioxidant properties of onion.

## CONCLUSION

Heat treatment showed deleterious effects on the antioxidant properties of onion and garlic. Conversely, the antioxidant activity of broccoli was enhanced through heat treatment. These findings suggest that thermal degradation of antioxidants may occur in some vegetables, while the antioxidant activity can increase through the improved extractability and matrix softening effects of heat treatment.

Freezing can improve the extractability of the antioxidant compounds of some vegetables like broccoli and garlic. On the other hand, frozen storage did not exhibit convenient conditions for preserving the antioxidant properties of cauliflower and onion. Additionally, the increased malondialdehyde levels of cauliflower may somehow have been a result caused by the increased oxidation process due to freezing.

Further research should be undertaken to establish what the most favorable vegetable consumption and storage patterns would be, to obtain the best benefit from the antioxidant compounds of vegetables.
